# Breastfeeding and Non-Communicable Diseases: A Narrative Review

**DOI:** 10.3390/nu17030511

**Published:** 2025-01-30

**Authors:** Maria Elena Capra, Valentina Aliverti, Arianna Maria Bellani, Martina Berzieri, Anna Giuseppina Montani, Gianlorenzo Pisseri, Tullia Sguerso, Susanna Esposito, Giacomo Biasucci

**Affiliations:** 1 Pediatrics and Neonatology Unit, Guglielmo da Saliceto Hospital, 29121 Piacenza, Italy; m.capra@ausl.pc.it (M.E.C.); g.biasucci@ausl.pc.it (G.B.); 2 Pediatric Clinic, Department of Medicine and Surgery, University of Parma, 43126 Parma, Italy; 3 Department of Medicine and Surgery, University of Parma, 43126 Parma, Italy

**Keywords:** breastfeeding, breast milk, non-communicable diseases, prevention

## Abstract

Introduction: Breastfeeding plays a fundamental role in newborns’ and infants’ health. Breast milk’s protective power against malnutrition and its positive effect on neurological and physical development are well established and are reflected in the policy statements of all major pediatric health entities. However, breastfeeding also plays an important role in the prevention of so-called non-communicable diseases, such as obesity, hypertension, dyslipidemia, and autoimmune diseases. Methods: This narrative review aims to analyze the effect of breastfeeding and breast milk on the development of non-communicable diseases, with a special focus on weight excess, dyslipidemia, allergy, and gastrointestinal diseases. This narrative review was carried out through three steps: executing the search, examining abstracts and full texts, and analyzing results. To achieve this, the databases PubMed, EMBASE, Scopus, ScienceDirect, Web of Science, and Google Scholar were explored to collect and select publications from 1990 to 2024 to find pertinent studies in line with this review’s development. The search included randomized placebo-controlled trials, controlled clinical trials, double-blind, randomized controlled studies, and systematic reviews. A total of 104 manuscripts were ultimately included in the analysis. Results: Breastfeeding is associated with a decreased vulnerability to early viral infections or chronic inflammatory conditions during preschool years, a reduced incidence of weight excess, and likely lower cholesterol concentration, besides having a small protective effect against systolic blood hypertension. Conclusions: Pediatricians must promote breastfeeding, support the mother–infant dyad, and consider breast milk as a real “health voucher” that can last lifelong. However, further studies are needed to better define the extent and duration of breastfeeding’s protective power in this context.

## 1. Introduction

Breastfeeding (BF) is pivotal in newborns’ and infants’ health. Breast milk’s (BM) protective power against malnutrition and its positive effect on neurological and physical development are well established and are reflected in the policy statements of all major pediatric health entities [1,2,3,4]. BM is a complex, ever-changing “biological system”, having protean purposes: to introduce neonates to enteral feeding and nourish them exclusively up to, ideally, the sixth month of life; to supply the neonate with readily-available immunologically active substances (over 250 different molecules, including cytokines, chemokines, immunoglobulins, cells, proteins, miRNAs, growth factors, and others [4,5]), along with their immature immune system development [6]; and to transfer the mother’s resident cutaneous bacterial population to the child, building the base for a healthy gut and skin microbiome [5].

The Developmental Origin of Health and Disease theory, sometimes called the “Barker hypothesis,” states that the relationship between genes and the environment causes lifestyle-related diseases to start at conception and progress through the embryonic, fetal, and neonatal stages. Any insult, either positive or negative, may have lifelong effects during the so-called “first thousand days” from conception [7]. Barker found that low birth weight and malnutrition in the early years of life are associated with the occurrence of ischemic heart disease in adulthood [8]. To survive environmental assaults brought on by inadequate nourishment, Barker’s hypothesis states that during pregnancy and the early postnatal period, an individual is programmed towards nutritional thrift [8]. This narrative review aims to analyze the effect of BF and BM on the development of non-communicable diseases, with a special focus on weight excess, dyslipidemia, allergy, and gastrointestinal diseases. We have decided to focus on these diseases as their incidence is constantly increasing in the pediatric population and their impact on developmental age is very important. To our knowledge, our narrative review is the first to specifically focus on the relationship between breastfeeding and targeted non-communicable diseases, such as weight excess and autoimmune diseases. BF offers mothers, children, and society immediate and long-term health, besides economic and environmental benefits. Political backing and monetary investment are required to safeguard, encourage, and support breastfeeding to realize these benefits [9].

## 2. Methods

This narrative review was carried out through three steps: executing the search, examining abstracts and full texts, and analyzing results. To achieve this, the databases PubMed, EMBASE, Scopus, ScienceDirect, Web of Science, and Google Scholar were explored to collect and select publications from 1990 to 2024 to find pertinent studies in line with this review’s development. The last search was carried out in December 2024. The search included randomized placebo-controlled trials, controlled clinical trials, double-blind, randomized controlled studies, and systematic reviews. The following combinations of keywords were used: “breastfeeding” OR “breast milk” OR “ human milk” AND “non-communicable diseases” OR “allergy” or “ autoimmune diseases” OR “cardiovascular disease” OR “dyslipidemia” OR “blood hypertension” OR “ type 2 diabetes mellitus” OR “ celiac disease” OR “overweight” OR “obesity” OR “weight excess” OR “microbiota”. We also performed a manual search of the reference lists of the selected studies. The search was limited to English-language journals and full papers only. Once the thorough search was conducted, the abstracts were reviewed to confirm that they pertain to the subject of interest. All copies were eliminated, and the abstracts of the leftover articles were evaluated to confirm that they meet the review inclusion criteria. As a result, the relevant studies concentrating on breastfeeding and its relationship with non-communicable disease were compiled and analyzed to create an integrated narrative review.

## 3. Breastfeeding (BF) and Allergy

Allergic diseases are prevalent in childhood and significantly affect morbidity and diminish quality of life [10]. In recent years, there has been a notable rise in the occurrence of conditions such as asthma, eczema, and allergic rhinitis [11]. Currently, more than 300 million individuals worldwide are impacted by asthma. Allergic rhinitis reportedly affects 10–30% of the global population. Both eczema and food allergies represent significant public health concerns, greatly influencing health-related quality of life [10]. The complexity and intensity of allergic diseases continue to escalate, particularly among children and adolescents, who experience the greatest impact from these conditions [12].

BF is believed to have a protective effect on allergic diseases’ development, though this topic is still debated. It is crucial for the optimal immune system development of infants, providing bioactive components and promoting the healthy establishment of the microbiome. Also, BM is the ideal food for babies, both for its immunomodulatory properties and its ability to protect against early infections. Since early infections are a significant risk factor for developing asthma and allergic diseases, the protective effects of BF may help shield against these conditions [10].

The intricate relationships among breastfeeding, the immune system of infants, and later allergic conditions are complicated and not completely comprehended. Numerous studies have investigated BF’s role in allergic disease development, yielding inconsistent results [13]. These discrepancies may be partly due to differences in methodological quality and study design. However, it is also likely that the true association depends on a combination of factors that are inconsistently evaluated across studies. These factors include the duration of BF, the timing of solid food introduction, population characteristics, cultural practices, and geographic regions [13,14].

### 3.1. Breastfeeding (BF) and Asthma

Asthma is a long-term condition characterized by recurring episodes of wheezing, coughing, and shortness of breath. These symptoms arise from increased airway sensitivity and inflammation of the airways. It is one of the more frequently reported chronic diseases of the respiratory tract [15]: the 2018 Global Asthma Report assumed that 339 million subjects suffer from asthma worldwide, and this figure is steadily increasing, especially among children. The development of asthma is influenced by multiple factors, including genetic predisposition and environmental factors such as early respiratory infections, antibiotic use, and exposure to smoking. Despite numerous studies on this topic, the relationship between BF and asthma remains challenging to clarify. This challenge is due to multiple factors, including recall bias regarding breastfeeding exposure, inconsistent management of confounding variables in statistical analyses, differing diagnostic definitions, and an overall scarcity of randomized controlled trials and robust cohort studies.

Some studies suggested that either any duration of BF or extended BF has a protective effect against asthma [16]. On the other hand, other studies showed no statistically significant association between BF and asthma [17].

It is recognized that bioactive substances in breast milk play a vital role in the development of newborns, and there are biologically feasible pathways through breastfeeding that may affect the development of asthma. A greater concentration of TGF-β1 has been linked to a reduced likelihood of wheezing by the age of one. The incidence of wheezing was found to decrease as TGF-β1 levels increased, which were derived from longer breastfeeding duration and higher concentrations of TGF-β1 in breast milk, in contrast to shorter breastfeeding duration and lower concentrations of TGF-β1 in breast milk. This relationship continues to hold significance even after accounting for factors such as sex, gestational age, maternal smoking, the presence of other children, maternal education, and maternal asthma. Given that wheezing is a risk factor for childhood asthma, this relationship is particularly significant [10].

However, BF duration does not seem to be linked to the development of lung function. In a prospective cohort study by Gorlanova et al., predominantly healthy, unselected children were monitored from birth to school age. At 6 years of age, their lung function was assessed using spirometry and body plethysmography. The research indicated that breastfeeding duration did not have a notable effect on lung function measures, such as airway obstruction, end-expiratory volume, and total lung capacity. These results were stable even after removing children who were diagnosed with asthma during their preschool years. Therefore, in contrast to earlier studies focused on asthma cohorts, such as the ISAAC Phase II study, which highlighted that breastfeeding was associated with higher predicted forced expiratory volume in one second in affluent countries only [11], this research observed no influence of breastfeeding on lung function in mostly healthy children. Research on the effect of BF on lung function varies in terms of age groups, risk factors, duration, and BF categories. Nonetheless, there is consistent evidence that BF does not positively affect lung function [18]. These findings reflected the ones in PROBIT studies, which similarly found no effect of BF on the development of lung function [19].

By integrating insights from these studies, we can hypothesize that the influence of BF on lung function development may be secondary, potentially mediated by a decreased vulnerability to early viral infections or chronic inflammatory conditions during preschool years, similar to those described in asthma [18].

### 3.2. Breastfeeding (BF) and Atopic Dermatitis and Allergic Rhinitis

There is limited-quality evidence suggesting that BF may be associated with a reduced risk of eczema within the first two years of life and a decreased risk of allergic rhinitis within the first five years [13].

Regarding eczema, this protective effect was observed in cohort studies only in children who had been exclusively breastfed for 3–4 months. After this age, the protective benefit of BF was no longer evident; in fact, there was limited evidence indicating a heightened risk. Subgroup analysis revealed that BF was linked to a higher risk of eczema in studies demonstrating poorer methodological design and extended recall periods of BF. These studies significantly impacted the combined estimates for eczema in individuals older than 2 years. BF seemed to provide enhanced protection against eczema in middle- and low-income nations, potentially because it guards against infections in early life, where viral rashes in infants could be mistaken for eczema. On the other hand, environmental elements could elevate the chances of eczema in affluent communities. Consequently, BF might only offer protection against the infantile eczema phenotype [13]. The PATCH Birth Cohort Study found that any form of BF, whether shorter or longer, exclusive or not, effectively reduces eczema up to the age of two years, unlike formula feeding [20].

Gorlanova et al. observed that a longer duration of BF was associated with a reduced risk of atopic dermatitis (AD) in girls: the risk of having atopic dermatitis was reduced by 4% for every week of BF duration [18].

A recent review suggests that human milk may protect against atopic dermatitis due to the presence of human milk oligosaccharides (HMOs), which support the infant gut microbiome and have immunomodulatory effects. The review highlights the diversity of HMOs, microbes, and metabolites among exclusively breastfed infants and states that specific microbial genes and products may be essential for this protective effect [21]. HMOs shape the infant’s gut microbiota and have immunomodulatory effects, which may protect from atopic dermatitis. The ability to metabolize specific HMOs may explain why human milk protects some infants from atopic dermatitis but not others. Human milk also contains immunoglobulins (Igs), such as secretory IgA and IgG, enzymes, and other protective factors, with variability in these components potentially influencing the development of atopy. Understanding these factors and the infant gut’s metabolic signatures is fundamental to understanding AD/eczema development. Further research is needed to elucidate the diversity of bacterial metabolism and bacterial metabolites involved in protection against AD/eczema in human infants [21,22].

Moreover, the protection of BF against allergic rhinitis in early childhood might also stem from the challenge of distinguishing between allergic rhinitis and viral rhinovirus infections in infants. The lower risk linked to BF in younger age cohorts might be due to BM-driven viral defense instead of protection against allergic rhinitis [13].

### 3.3. Breastfeeding (BF) and Food Allergies

BM has active immune components, including cytokines, inflammatory agents, signaling substances, and soluble receptors that could lower the likelihood of allergic conditions. The American Academy of Pediatrics indicates that the connection between the duration of breastfeeding and the occurrence of food allergies in early childhood remains uncertain [23].

BF appears to protect against several common childhood allergic diseases, but not against food allergies. This discrepancy may be attributed to several factors, including the limited number of studies on food allergies and potential inaccuracies in measuring outcomes. Furthermore, the delayed onset of the food allergy epidemic and changes in BF guidelines over time could have obscured any potential link between BF and food allergies in recent research [13]. Grimshaw et al. distinguished between IgE-mediated and non-IgE-mediated food allergies, finding that an earlier introduction of complementary feeding (CF) was significantly associated with non-IgE-mediated, but not with IgE-mediated food allergies, by the age of 2 years [24]. Venter et al. noticed that early (before 16 weeks) compared to late (after 16 weeks) CF introduction was associated with a significantly reduced risk of food allergies at both 1 and 3 years of age [25,26].

These two large birth cohort studies suggested that the early introduction of solid foods either has no impact on the development of food allergies or might have a negative effect. However, these studies encompassed both IgE-mediated and non-IgE-mediated food allergies, with a relatively small number of infants having IgE-mediated food allergies [24,25].

Lachover-Roth et al. noted that infants who were breastfed exhibited notably elevated rates of IgE-mediated food allergies in their first year, possibly as a result of substances consumed by the mother that were transferred into the BM. Ultimately, although the function of BM in the emergence of food allergies is not well understood, it is clearly recognized that the introduction of solid foods is an essential time for infant growth. Current guidelines recommend starting solid food introduction between 4 and 6 months while continuing BF. The introduction of potentially allergenic foods, such as peanuts and eggs, while the infant is breastfed may help reduce the risk of allergies. Delaying the introduction of these foods beyond six months does not appear to lower allergy risk [27].

In conclusion, despite the ongoing debate in the literature and the inability to definitively demonstrate a protective effect of BF against the development of allergic diseases, it remains advisable to promote exclusive BF for the first six months of life. This recommendation is based on the well-documented and widely acknowledged nutritional and immunological benefits provided by BM [28]; indeed, BM’s protective effect against asthma, eczema, and allergic rhinitis seems strongest in early life, possibly due to protection from viral diseases, misdiagnosis, or effects on specific phenotypes [13]. BF’s preventive effects appear to diminish with age, especially in children with a family history of allergies. The GINIplus data support BF for allergy prevention due to its beneficial effects in early life. Future studies are needed to confirm potential long-term protection against allergic diseases in children with a low family risk of allergies [29].

Further studies are needed to shed light on this topic, aiming to clearly define factors such as the definition of “exclusive BF”, the duration of such BF, the timing of introducing solid foods, the characteristics of the analyzed population, and the definitions of the diagnosis itself to minimize biases as much as possible.

## 4. Breastfeeding (BF) and Autoimmune Diseases

BM can help transfer the mother’s resident cutaneous bacterial population to the child, building the base for a healthy gut and skin microbiome [5]; therefore, it might have an impact on the development of autoimmune diseases. The effects of BF on the development of autoimmune diseases have been studied to different extents with varying success rates.

### 4.1. Breastfeeding (BF) and Type 1 Diabetes Mellitus

Many studies have been conducted in the past decade to investigate the association between BF and type 1 diabetes mellitus (T1DM). A meta-analysis conducted in 2012 by Cardwell et al. analyzed data from 43 observational studies involving 9874 subjects, finding a significant reduction in T1DM incidence in children exclusively breastfed for at least 2 weeks, compared with those breastfed for less than 2 weeks (0,86 O.R.; 95% CI 0.75–0.99; *p* = 0.04), only in selected studies with low heterogeneity (*I*^2^ = 0%; heterogeneity; *p* = 0.44), but no significant protective effect in longer durations of exclusive BF or any duration of nonexclusive BF was found. The authors remarked that it was not possible to discern more precisely which duration of exclusive BF, beyond 2 weeks, maintained a protective effect [30]. Güngör et al. investigated the correlation between ever vs. never BF, the duration of any BF, and the duration of exclusive BF, and T1DM incidence. The results showed limited evidence of an association between never being fed BM and T1DM, moderate evidence that a shorter duration of any BM feeding is associated with the incidence of T1DM, and limited evidence that a shorter duration of exclusive BM feeding is associated with a higher incidence of T1DM [31]. Lund-Blix et al. conducted a study on 155,392 Scandinavian children, following them from birth and administering questionnaires to their parents at 6 and 18 months of life: the data from this cohort study showed that never-breastfed vs. ever-breastfed children had a two-fold higher risk of developing T1DM. Interestingly, no difference between exclusive and any BF, or shorter and longer duration of BF, was found with regard to the incidence of T1DM [32,33].

Lund-Blix et al. screened 50,000 Scandinavian neonates for high-risk HLA genotypes, finding 908 neonates with a genetic predisposition for T1DM (of which 726 completed the entire follow-up), and followed up by taking blood samples and administering questionnaires at 3, 6, 9, and 12 months of age and then annually. The authors found that BF for 12 months or longer was associated with a lower incidence of T1DM and a lower percentage of progression from islet autoimmunity to T1DM, but no protective effect against the development of anti-islet immunity was detected [34]. As highlighted by the qualitative review conducted by Vieira Borba et al. and by many other studies, the available literature so far, is suggestive of a significant protective effect of BM and BF on T1DM, but, due to the nature of the issue, a lot of research is still needed to understand what duration and what exclusivity of BF are protective and in which populations. Large, prospective birth control studies are still ongoing and, hopefully, they will provide us with interesting information on this topic [5].

### 4.2. Breastfeeding (BF) and Other Autoimmune Diseases

In 2022, Holtz et al. conducted a meta-analysis of 15 studies and showed a significant protective effect of any BF vs. no BF against the development of multiple sclerosis (MS), and this correlation remained when stratifying subgroup analysis by study design and when restricting the analysis to low risk of bias studies [35]. Conradi et al. and Ragnedda et al. found an increased incidence of MS in children when BF duration was lower than 4 months [36,37]. On the contrary, the 2017 case-control study by Graves et al. analyzed various maternal and neonatal exposures (including BF) to MS incidence and found no correlation between MS incidence and BF [38].

The relationship between BF and the development of Juvenile Idiopathic Arthritis (JIA) is unclear: some studies found a protective effect of any or longer BF towards JIA development [39,40], whereas others found no association [41,42,43]. This discrepancy may be due to unaccounted-for confounding factors, heterogeneity in study populations and methods, or different statistical analyses. Moreover, larger prospective studies designed to control for possible confounders are needed to understand the true role of BF in the development of JIA.

Chen et al. conducted a meta-analysis to investigate the relationship between BF and Rheumatoid Arthritis (RA), and they found a statistically significant negative correlation between BF (1–12 months and >12 months) and the development of RA but no correlation between duration of BF and the reduction in RA incidence [44]. Alotiby et al. and Kindgren et al. examined the relationship between RA and BF; both studies showed that exclusive BF might have a protective effect against RA, as compared with exclusive formula feeding and mixed feeding [33]. In 1990, Fort et al. carried out a case-control retrospective questionnaire-based study to investigate the relationship between Hashimoto Thyroiditis (HT) and BF: they obtained a history of the feeding practices of 59 children with HT, 76 healthy siblings, and 54 healthy unrelated controls and found no difference in BF prevalence or duration in the three groups. However, this study found a much higher prevalence of early exposure to soy-based formula in the HT group compared with both healthy control groups [45]. As HT is one of the most common autoimmune diseases in the pediatric age, further research is needed to ascertain whether or not BF has any correlation with AT, also due to its potential clinical value [46].

## 5. Breastfeeding (BF) and Cardiovascular Diseases

Cardiovascular diseases (CVDs) continue to be the leading cause of death and illness in industrialized nations [47,48]. Numerous studies indicate that early atherosclerosis can be identified in childhood, and its development is influenced by various risk factors such as metabolic changes, hypertension, chronic inflammation, and possibly existing structural or functional heart disorders [49].

Inflammation can augment CVD risk through the so-called “dyslipidemic atherogenic triad” characterized by elevated triglycerides, low high-density lipoprotein (HDL) cholesterol plasma levels, and elevated low-density lipoprotein (LDL) cholesterol plasma levels [50].

Prevention is feasible and desirable starting from childhood to prevent risk factors (primordial prevention), to recognize and subsequently manage risk factors in childhood (primary prevention), and to mitigate the risk of further incidents in individuals who have already encountered coronary artery issues (secondary prevention) [50].

**Figure 1:**
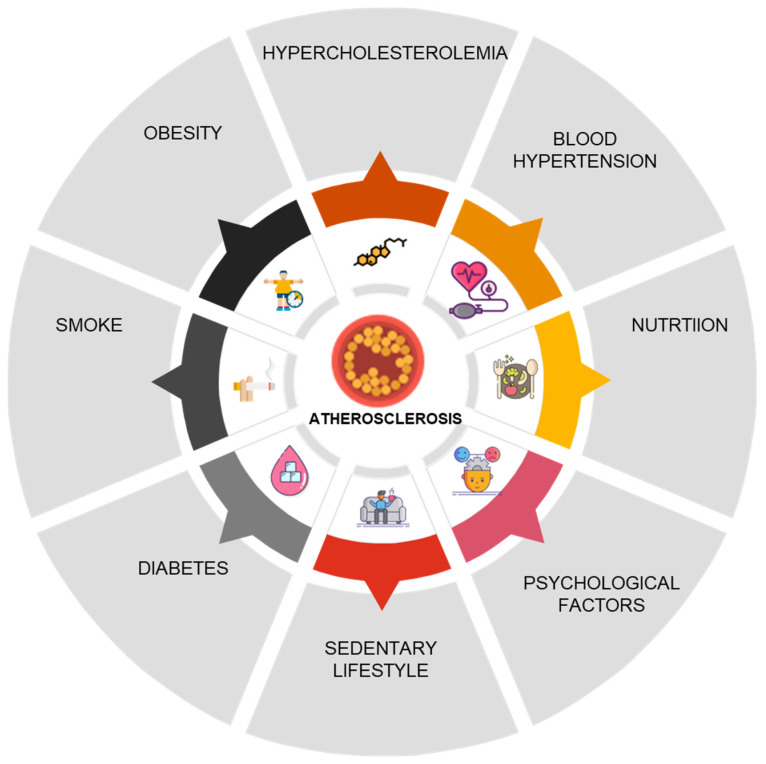
Risk factors that can influence atherosclerosis, modified from [57].

Primordial prevention with BF should start immediately after birth: indeed, early exposure to BM can have protective effects, thus reducing the development of CVDs in adulthood [51,52]. Antibodies, stem cells, hormones, HMOs, growth factors, and enzymes found in BM may promote the formation of cardiovascular tissue in the neonatal and infant phases [51]. Adiponectin and vascular endothelial growth factor (VEGF), which may be extremely important for the cardiovascular system, are also found in BM [51]. Adiponectin may cross the intestinal barrier and plays a variety of pleiotropic effects in controlling inflammatory and metabolic pathways [53], whereas VEGF is a major modulator of angiogenesis and vasculogenesis [4]. Furthermore, both cardiac and vascular cells are protected by adiponectin [54]. BM also contains lactoferrin, which is a component of the innate immune system that exerts antibacterial activity in infants. The greatest concentration of lactoferrin is present in colostrum, although it remains in breast milk during the entire lactation period. Lactoferrin also possesses anti-inflammatory qualities, and its receptors can be found in various tissues throughout the body, such as the heart and vascular system [55]. Various kinds of stem cells are also distinctly present in BM. Significantly, BM stem cells have demonstrated the ability to differentiate into various cell types, such as cardiomyocytes [56]. HMOs are taken in by infants and have significant functions in immune and inflammatory regulation [51]. According to the historical INTERHEART STUDY, cardiovascular disease can be influenced by numerous risk factors that are summarized in Figure 1 [57].

### 5.1. Weight Excess

The World Health Organization (WHO) defines obesity as “one of the most serious challenges of the 21st century” [49] and estimates that in 2022 overweight affected around 37 million children under the age of 5 globally, and that over 390 million children and adolescents aged 5–19 years were overweight worldwide. Infancy is an important stage of life to prevent overweight in childhood. Excess weight raises the likelihood of various non-communicable diseases, such as diabetes, cancer, and cardiovascular diseases. It has been proposed that BF might help to avoid the onset of overweight/obesity, not just during the early years of life but also in later years. In 2001, Gillman et al. conducted a survey of 8186 girls and 7155 boys, aged 9 to 14 years, discovering that those primarily fed breast milk during the initial 6 months of life had a reduced prevalence of overweight 9 to 14 years later, following the correction of variables indicating social, economic, and lifestyle characteristics, such as age, sex, sexual maturity, energy intake, television viewing time, physical activity, and mother’s body mass index [58]. Compared with subjects who had been predominantly fed infant formula, the estimated relative risk reduction was approximately 22% [58]. Horta et al. discovered 33 studies that discussed the impact of breastfeeding (BF) on the rates of overweight and/or obesity. They found that children with longer BF duration have a 10% lower prevalence of being overweight or obese [59]. However, socioeconomic status-related residual confounding provides an additional explanation for the results. This methodological issue should be taken into account when evaluating the evidence about the long-term effects of breastfeeding [59].

In conclusion, current evidence suggests that supporting BF holds promise in reducing the burden of obesity in this community, and the longer the duration of BF, the greater its preventive effectiveness.

**Figure 2:**
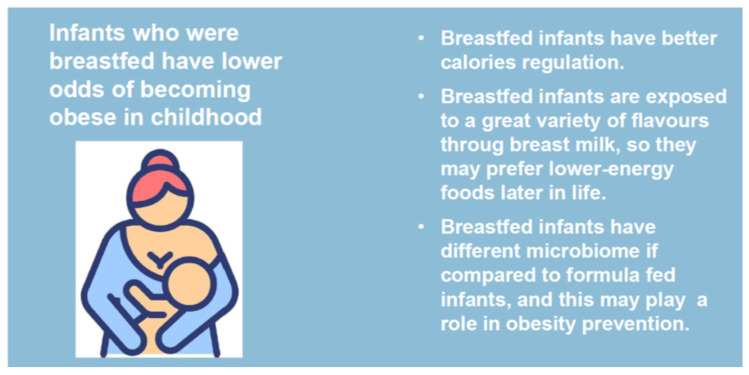
Effects of BF on weight excess prevention.

BF’s protective role against weight excess development can act through various mechanisms, including the optimal nutrients provided by BM and its lower protein and higher fat content compared with formula milk [59], which in turn may predispose to increased early adiposity. Furthermore, BM contains the hormones adiponectin, ghrelin, and leptin, which may have a beneficial effect on the deposition of body fat [58,60,61,62]. Additionally, compared to baby formula-fed babies, breastfed babies might have greater control over how much milk they drink [63], which could help them better control their energy intake later in life. According to other research, BF might potentially have modulatory effects on the degree of expression of genes that predispose people to obesity [64,65]. Beneficial effects of breastfeeding in terms of weight excess prevention are summarize in Figure 2.

### 5.2. Blood Hypertension

In adult subjects, blood hypertension has a strong influence on the risk of coronary heart disease and stroke. It has been postulated that factors operating early in life (in utero, infancy, and childhood) may influence the value of blood pressure in adulthood [61], and that hypertension during childhood can have unhealthy consequences [62]. Naghettini et al. [62] evaluated 519 children aged 3 to 10 years, and they observed the protective effect of longer BF (>6 months) on the development of blood hypertension later in life. Data from 933 children at 2, 6, and 24 months were gathered by De Jonge et al. [63], who found no variations in blood pressure, fractional shortening, or heart architecture between breastfed and non-breastfed children. In a survey of 377 Japanese children aged seven, Hosaka et al. [61] highlighted that nursing had a preventive effect against childhood high blood pressure, even if exclusive breastfeeding may not be required to obtain the long-term blood pressure advantages. Järvisalo et al. [64] assessed cardiovascular risk variables and endothelial function in 1667 adults between the ages of 24 and 39. They discovered that adult males who were breastfed had better brachial endothelial function than those who were fed formula. In a cross-sectional study on health and lifestyle, Holmes et al. [65] looked at a group of schoolchildren between the ages of 12 and 15 who were followed into young adulthood until they were between the ages of 20 and 25. They found no significant differences in anthropometric measurements, blood pressure, or plasma lipid profiles between adults who were breastfed and those who were not. A cohort of 9377 subjects born within a week in England, Scotland, and Wales were periodically followed up from birth through adulthood by Rudnicka et al. [66] in 1958. Their findings demonstrated that there was no significant long-term protective effect of BF for more than a month on other CVD risk factors in adult life.

These findings are consistent with a small protective effect of BF against systolic blood pressure mostly in childhood, but residual confounding factors such as growth environment and socioeconomic and demographic characteristics cannot be ruled out [59].

### 5.3. Hypercholesterolemia

Dyslipidemia is considered an important modifiable risk factor for CVD. Nowadays, the prevalence of dyslipidemia in children and adolescents is epidemically increasing, and this emphasizes the importance of early intervention during childhood and adolescence to prevent the development of atherosclerotic CVD [67].

BM has a higher cholesterol content if compared with formula milk but, as demonstrated by Owen et al., higher intakes of cholesterol in the first months of life can help down-regulate hepatic hydroxy-methylglutaryl coenzyme A (HMG-CoA), thus reducing cholesterol synthesis: BF may have a long-term programming effect on blood cholesterol levels; therefore, adults who had been breastfed will have lower total cholesterol (TC) and low-density lipoprotein cholesterol (LDL-C) if compared with those who had been formula fed [59].

Previous research has indicated that feeding infants can influence long-term alterations in cholesterol metabolism [68], and initial BF (particularly when exclusive) may be associated with lower blood cholesterol concentrations later in life [68], even if other studies [59] show that the beneficial effect of BF on blood TC in adulthood is smaller than that estimated by earlier reviews [64,65,66,69].

A study conducted in 11.0 ± 0.5-year-old children shows a statistically significant association between triglyceride levels and breastfed patients, irrespective of the child’s BMI, socio-demographic, and lifestyle characteristics. This study did not find any difference between TC, LDL, and non-HDL levels among breastfed and formula-fed children. A positive association between HDL-C levels and BF was detected, even if this association was attenuated after adjustment for the child’s BMI [68]. Recently, Li et al. enrolled 12,110 Chinese children and adolescents aged 5–19 years and analyzed serum TC, LDL-C, and HDL-C levels. They found that in those children who had been breastfed for more than 12 months the TC, LDL-C, HDL-C, and TC/HDL-C ratio decreased by 6.225 (95% CI: −8.390, −4.059), 1.956 (95% CI: −3.709, −0.204), 1.273 (95% CI: −2.106, −0.440) mg/dL, and 0.072 (95% CI: −0.129, −0.015), respectively, compared with those who were not breastfed [70]. L.L. Hui et al. showed that exclusive BF in the first 3 months of life is related to lower serum TC and LDL-C levels in 17.5-year-old patients [71]. Similar results were obtained in preterm patients: the ratio of LDL to HDL cholesterol was significantly lower in adolescents who had been fed with banked BM compared with those who had received preterm formula (mean difference 14% lower) [72].

Li et al. looked into the conceivable mechanism behind the positive effects of BM. They found that the rate of cholesterol synthesis was inversely connected with the amount of cholesterol consumed through diet: BM has more cholesterol than formula milk (90–150 mg/L versus 0–4 mg/L) [70], which inhibits the production of endogenous cholesterol. Furthermore, BM contains a lot of bioactive substances that have antioxidative properties; additionally, the structure and composition of phospholipids in BM and formula milk are different, particularly in terms of molecules that are easier to absorb [70].

### 5.4. Glucose Metabolism

Over the past ten years, the amount of children impacted by type 2 diabetes mellitus (T2DM) has increased significantly. Findings from the National SEARCH for Diabetes in Youth study indicated that the prevalence of T2DM among individuals aged 10 to 19 years was 12.5 cases per 100,000 during 2011 and 2012, showing a yearly rise of 7.1% [73]. Limited evidence exists regarding the connection between BF and the occurrence of T2DM. A 2022 study linked BF to a reduction in ACAC-B gene expression, which provides protection against diabetes, along with an increase in PPAR-γ gene expression. No proof was discovered regarding BF’s impact on the expression levels of the BDNF, LXR-α, and PTEN genes among children who were ever breastfed compared to those who were never breastfed [74]. This condition might result from compounds present in BM that could induce epigenetic changes in infants in their early development [75]. A subset of 710 teenagers from Hong Kong’s “Children of 1997” birth cohort was examined at age 17: results revealed that teens who were breastfed during the first 3 months of life had reduced fasting insulin and HOMA IR (*p*-for-trend < 0.05), though not reduced fasting glucose [76].

### 5.5. Cardiac Morphology

There is evidence that there is a beneficial association between BM and cardiac morphology in adult life in subjects born prematurely [77]. Lewandowski et al. tracked 102 subjects from a group of 926 preterm infants who initially participated in a randomized controlled trial assessing postnatal milk-feeding strategies from 1982 to 1985, across five various centers in the UK. Out of these, 30 were assigned to exclusive breast milk and 16 to exclusive formula feeding. For the comparison group, they enlisted 102 healthy subjects who were born at term. They evaluated cardiac structure and function using MRI and found that preterm infants who were solely breastfed in infancy had higher left and right ventricular end-diastolic volume index (+9.73%, *p* = 0.04 and +18.2%, *p* < 0.001) and stroke volume index (+9.79%, *p* = 0.05 and +22.1%, *p* = 0.01) compared to preterm infants who were exclusively fed formula [78].

All the studies available to date support the WHO recommendations on BF. Nowadays, available studies show a strong association between the protective effect of BM and obesity. However, the correlation between BF and the development of dyslipidemia or alterations in glucose metabolism needs further investigation.

Further studies are needed, especially to evaluate the effectiveness of BF in adulthood. Moreover, there is a need for prospective studies focusing on the type and duration of BF, as in most studies conducted to date, these data were collected retrospectively, thus providing limited information.

Another limitation in the various studies is that the lifestyle of the patients in the post-BF period is seldom taken into account, so further research would be needed to adjust the results obtained to the type of diet and physical activity of the patients. Further future studies should also investigate the difference in the long-term effectiveness of BF on metabolism, depending on the mode of human milk delivery.

## 6. Breastfeeding and Microbiota

In addition to supplying essential nutrients for healthy growth, BM also provides commensal bacteria that further enhance the infant’s health [79]. The bacterial communities present in BM significantly impact infants’ health and development, contributing to the establishment of the gut microbiota in the early years of life, with positive effects that last throughout adulthood.

An adult microbiome that is comparatively stable is established by early microbial colonization of the infant’s gut. The earliest days and weeks of life have a significant impact on how the immune system, gastrointestinal tract, and adult microbiome develop. Conditions like obesity, food allergies, and inflammatory bowel diseases can be influenced by early environmental exposures to the microbiome [80].

While BM was once considered a sterile fluid and the microbes found in it were viewed as contaminants, it is now recognized that BM harbors a unique microbiome. The characteristics of this microbiome can be influenced by various factors such as the mother’s diet, age, metabolic state, genetics, and family lifestyle, although quantifying the contribution of each is complex [79].

Therefore, the development and maturation of the intestinal microbiota are dynamic and non-random processes characterized by both positive and negative interactions among major microbial taxa. These processes are influenced by various perinatal conditions such as mode of delivery, feeding type, and antibiotic use [81].

Numerous studies indicate that the composition of the infant gut microbiome is crucial for immune system development, especially in the first three months of life. Research has shown that early gut “dysbiosis” may be associated with immune dysregulation, both acute and chronic, leading to common conditions like allergies, asthma, diabetes, obesity, irritable bowel syndrome, and Crohn’s disease, in both childhood and adulthood [82].

The development of the intestinal tract, immune system programming, and related metabolism all depend on microbial colonization of the human gut. The microbiota and the host must communicate constantly in order to coordinate these physiological processes. Long-term physiological effects and health issues can result from gut “dysbiosis” that interferes with this conversation [79].

Through direct exposure to BM microbiota and indirect exposure to BM-derived factors and bioactive compounds that support bacterial growth and metabolism, BF affects the development of neonatal gut microbiota [79]. According to recent research, BM influences the child’s immune development in addition to offering passive protection by transferring a variety of immunological and microbiological factors from the mother. These early imprinting experiences have long-lasting consequences in adulthood and are essential for immunological and metabolic homeostasis [83].

Despite infant formula milk being designed to closely resemble human milk, the intestinal microbiome of formula-fed infants significantly differs from that of breastfed infants [84].

Numerous factors, including maternal diet, genetics, health status, delivery method, and demographic or environmental differences, can be responsible for variations in BM microbiota [85,86]. Numerous investigations have looked into how these factors affect BM microbiota. In their study of the impact of gestational age, delivery method, and lactation phase on BM microbiota, Khodayar-Pardo et al. discovered that, in comparison to vaginal delivery, cesarean section delivery was linked to higher total bacterial concentrations early in lactation, with significantly higher levels of Streptococcus spp. and lower levels of Bifidobacterium spp. Similar to this, Hermansson et al. found that the mode of delivery and intrapartum antibiotic exposure significantly altered the microbial composition of milk [79].

The intestinal flora of breastfed infants is typically dominated by *Bifidobacterium* and *Lactobacillus* species, whereas formula-fed infants exhibit a more diverse intestinal microbiota, with a higher presence of *Escherichia coli*, *Clostridium*, and *Bacteroides* [87].

*Bifidobacteria* typically dominate the intestinal microbial community in 3–4-month-old infants. These bacteria are likely enriched in the guts of breastfed infants due to the variety of oligosaccharides present in BM, which also serves as a source of live *Bifidobacteria*. Breastfed infants have been shown to host a more complex and abundant *Bifidobacterium* microbiota compared with formula-fed infants [81]. *Bifidobacteria*, as a dominant group, contribute to producing metabolites that allow other bacterial populations to permanently colonize the intestine later on [81].

Variations in the *Bifidobacteria* population among infants have led to comparisons on the status of the immune system between those with an abundance of these bacteria and those failing to expand. Lack of *Bifidobacteria* has been associated with increased neutrophils, basophils, plasmablasts, and memory CD8+ T cells, indicating immune activation both in innate and adaptive processes. In contrast, infants with abundant intestinal *Bifidobacteria* showed higher frequencies of non-classical monocytes, often considered anti-inflammatory, and regulatory T cells expressing CD39 receptor, a highly suppressive Treg subgroup [88,89].

Additionally, plasma proteins differed between these groups: infants lacking *Bifidobacteria* had elevated levels of TNF-α and IL-17A, key mediators of intestinal inflammation, as well as Th2 cytokines IL-13 and IL-1α, acting as danger signals released by necrotic cells and synergizing with TNF-α in a variety of cells. Conversely, infants with abundant *Bifidobacteria* showed higher levels of Treg-associated cytokines such as IL-27 and IL-10 and endogenous inhibitors of IL-1, which likely regulates innate IL-1β-mediated responses. Surprisingly, IL-6 was increased in infants with abundant *Bifidobacteria* [82].

Specifically, metabolites produced by Bifidobacterium have been demonstrated to influence pathogen-induced inflammation via the aryl hydrocarbon receptor (AhR) and NRF-2 pathways [90]. Research shows that the genes for HMO utilization expressed by Bifidobacteria and other helpful microbes in breastfed infants are linked to lower systemic inflammation and reduced Th2 and Th17-type responses [82]. BM contains abundant HMOs, complex glycans indigestible to the infant but representing the third most abundant component of human milk after lactose and lipids, nourishing bacterial communities in the infant gastrointestinal tract [79].

Maternal energy expenditure to create these complex sugars is justified by the selective nutritional advantage they offer to HMO-metabolizing beneficial microbes, with evolutionarily important functions in neonates. *Bifidobacterium longum* subsp. *infantis* (*B. infantis*) is one such strain adapted to metabolize HMOs and is common in breastfed infants in low-incidence countries for immune-mediated diseases [79].

HMOs function as prebiotics, serving as metabolic substrates that encourage the proliferation of beneficial microorganisms in the gut microbiome of infants. Along with fostering the development of beneficial bacteria, HMOs influence the reactions of intestinal epithelial cells and hinder pathogen attachment to the intestinal epithelium. Research indicates that beneficial microbes such as Bifidobacterium spp. adjust to metabolize HMOs in the gut of infants, simultaneously inhibiting the proliferation of potentially harmful bacteria [79].

HMOs can also alter gene expression related to commensal metabolic functions, thereby impacting the release of metabolites that may influence growth and inflammation [80]. Human BM contains a higher percentage of microRNAs compared with infant formulas. BM microRNAs play immune and metabolic roles, with a primary role in aiding the development of the infant’s immune system [79].

Secretory IgAs are the most abundant immunoglobulins in human milk, with higher concentrations early in lactation, representing 90% of all antibodies present. These antibodies are specific to numerous intestinal pathogens and commensals, facilitated by selective migration of B cells from mucosal membranes to the mammary gland. They provide neonates with excellent mucosal passive immunity and influence the composition of the intestinal microbiota, promoting colonization of beneficial bacteria such as *Bacteroides*, *Bifidobacterium*, and *Lactobacillus*. The absence of secretory immunoglobulins-A (IgAs) has been associated with alterations in the intestinal microbiome and gene expression in the intestinal epithelium, increasing susceptibility to intestinal inflammation later in life [80].

Microbial colonization can establish an immune–microbial balance or lead to varying degrees of intestinal and systemic inflammation, disrupting immune cell regulation [82].

This underscores the importance of early colonization of the microbiome during a critical window of immunological development, offering opportunities to integrate the intestinal microbiome with long-term health benefits for the child [82].

Recent studies suggest that altering intestinal microbiome balance during a critical period may have lasting consequences on immune-related pathologies. Cross-dialogue between commensals and mucosal surfaces of the body, facilitated by hos–-microbiome interactions in the early days of life or even during pregnancy, is crucial for immune defense development. This cross-dialogue suggests that disease risk is programmed in early life stages, including the prenatal period [81].

To summarize, the early development of the intestinal microbiome is a complex process with long-term implications for health. Although a number of factors affect microbial assembly, BF is thought to be one of the most important ones for the composition of the intestinal microbiome in infancy.

Differences in microbial communities between breastfed and formula-fed children are consistent and considered to mediate the relationship between BF and reduced risk of numerous diseases in early life [91]. The connection between human BM, intestinal microbiome, and chronic/atopic diseases is an active area of scientific research [91,92].

## 7. Breastfeeding (BF) and Gastrointestinal Diseases

### 7.1. Celiac Disease

Celiac disease (CD) is a multifactorial immune-mediated systemic disorder caused by a complex interaction between genetic and environmental factors. Genetic predisposition plays a major role in the development of CD. However, it has been observed that CD patients who were breastfed have a better long-term health status. This benefit could be attributed to the longer period between the introduction of gluten and the development of the disease, the lower incidence of gastrointestinal infections in breastfed children, and the prevention of gut “dysbiosis” [5]. BM can therefore be considered an influential environmental factor in this disease. Several cross-sectional studies have examined infant feeding by comparing it with the manifestations of CD [93]. BF has been shown to offer independent protection against CD if infants are breastfed during the introduction of gluten-containing foods. This protective effect is even stronger in children who continue to be breastfed after the introduction of gluten [94]. Biologically, the presence of BM during gluten introduction can increase the likelihood of developing oral tolerance to the relevant antigens [94]. During lactation, the mammary gland and the baby’s gut form an immunological dyad. BM contains several defensive factors, such as secretory IgA antibodies, the free secretory component, lysozyme, and lactoferrin, which protect the baby’s mucosal surfaces. This protection is crucial in the so-called ’window of opportunity’ before the baby’s immune system is completely developed. In addition, BM contains cytokines and leukocytes that can influence the baby’s immune system in a more sophisticated way, with potential long-term effects. The intestinal immune system can respond to foreign antigens in two distinct ways. The first mode is a tolerant-type response, often called oral tolerance, which depends on the amount of antigen introduced and is the default response of the intestinal immune system. The second mode is an aggressive response, useful for fighting viruses and bacteria.

The CD results from an inappropriate immune response to gluten in the gut. This response is characterized by a delayed hypersensitivity reaction dominated by the production of interferon γ by T cells. Gluten-responsive T cells primarily recognize modified (deamidated) gluten peptides by the enzyme tissue transglutaminase (TG2). This enzyme is induced by tissue destruction and inflammation. Intestinal infections increase TG2 expression, promoting the generation of deamidated gluten peptides. Simultaneously, this environment instructs the intestinal immune system to react aggressively. Therefore, an intestinal infection can trigger the harmful T-cell response perpetuated by gluten in CD. In addition, gastrointestinal tract infections can increase the permeability of the intestinal mucosa, allowing gluten to pass into the lamina propria, thus exacerbating the immune response [93].

The simplest model to explain the BM protective effect in CD is its ability to protect against intestinal infections. However, more complex models can be hypothesized, such as the activation of cytokine networks or idiotypes by milk components. These more sophisticated models need further study and investigation to be understood fully [93]. Thus, due to its immunomodulating effect, BF can modify the immune system’s response to antigen exposure [94]. A meta-analysis and systematic review, including studies conducted between 1966 and 2004, found that breastfed infants had a 52% lower risk of developing CD compared with those not breastfed at the time of gluten introduction [5]. Case-control studies showed that children with CD were less likely to have been breastfed or breastfed for a shorter period than children without CD [95]. The reduction in the risk of developing CD also relates to BF duration. A recent review of six studies showed that the risk of developing CD was significantly reduced in infants who were breastfed at the time of gluten introduction, compared with infants who were not breastfed. However, although BF may delay the onset of CD symptoms, it may not offer permanent protection against the disease [96]. In a case-control study, Ivarsson et al. investigated whether BF and the method of introducing gluten into the diet influenced the risk of CD in a sample of 627 Swedish celiac children compared to 1254 unaffected ones. They found that the risk of developing CD was lower in children who were breastfed when gluten was introduced into their diet [94]. Thus, epidemiological studies suggest that early infant feeding practices may play a role in the environmental risk factors contributing to the development of CD, indicating that BF may offer protection against the development of CD. However, we cannot establish whether BF provides permanent protection against the development of CD or only delays symptom onset [93]. In conclusion, BF during gluten introduction into the diet and a longer BF duration may offer protection against the development of CD. Nevertheless, further long-term studies are needed to determine the relationship between BF and CD [93].

### 7.2. Inflammatory Bowel Diseases

Inflammatory bowel diseases (IBDs) are chronic inflammatory conditions of the gastrointestinal tract, whose pathogenesis remains partly unknown. However, it is believed that a dysfunctional host immune response to gut flora, in genetically susceptible individuals, can be implied in their etiology [5]. The evolution of epidemiology over time and in different geographical areas suggests that environmental factors are crucial in promoting or modifying IBD expression. Critical events during childhood, such as birth, BF, and exposure to antibiotics, as well as experiences during growth, are considered potential risk factors for IBDs. Furthermore, it is recognized that the gut microbiota plays a central role in the development and exacerbation of inflammation in these forms of the disease. Although an individual’s genetics partially influence the composition of the gut microbiome, environmental exposures from childhood to adulthood continue to shape the structure and function of the gut microbiome. This can lead to a state of “dysbiosis”, thus contributing to the occurrence of IBDs [97]. The highest incidence of IBDs occurs in early adulthood, indicating that early exposures may force future susceptibility [5]. BM can influence the immune tolerance and bacterial colonization of the infant’s gut. Several studies have indicated that BF protects against IBDs, especially in the early stages of its onset. However, research conducted to test this hypothesis has not yet produced definitive or conclusive results [5]. Many studies did not precisely define the duration and exclusivity of BF. Moreover, it is not clear whether the studies compared exclusive to non-exclusive BF, or whether they compared non-exclusive breastfeeding to bottle-feeding. Consequently, it is not possible to establish with certainty whether the risk of developing IBDs is related to the absence of BF or the presence of bottle feeding [24]. However, considering that BF’s immunomodulatory effect offers protection against several diseases of autoimmune pathogenesis, it is plausible to postulate a similar protective effect against IBDs. Breastfed infants, during the development of the immune system, may acquire oral tolerance to specific microflora and food antigens, which may play a role in the pathogenesis of IBDs [98].

In the Canadian Healthy Infant Longitudinal Development (CHILD) cohort study, fecal samples from 4-month-old infants were examined to explore the link between gut microbiota makeup and several perinatal factors, such as the infant’s diet [99]. Actinobacteria and Firmicutes were the predominant phyla found in both groups, with reduced bacterial diversity noted in breastfed infants compared to those fed with formula. Infants fed with formula exhibited a greater presence of Peptostreptococcus, especially Clostridium difficile, which is linked to atopic reactions and allergic sensitization [99]. Intrapartum antibiotics during cesarean and vaginal deliveries are linked to “dysbiosis” in the gut microbiota of infants, and breastfeeding alters some of these impacts [100]. In animal research, Il10-/- mice showed lower levels of systemic TNF and IFNγ and less severe histological gut inflammation when given mouse breast milk [101]. Since 1961, a potential link between BF and the development of ulcerative colitis has been proposed [97]. A meta-analysis of 17 pertinent studies investigated the relationship between BF and IBDs, revealing a significant negative correlation with both Crohn’s disease (OR 0.45, 95% CI 0.26–0.79) and ulcerative colitis (OR 0.56, 95% CI 0.38–0.81) [98]. A systematic review concentrating on pediatric IBDs similarly showed a significant inverse relationship with early-onset disease [102]. The data supports the hypothesis that early or late changes in the gut microbiota, along with not being breastfed, could be significant risk factors for IBDs by affecting the development of the gut microbiome [97].

The relationship between BF and the analyzed non-communicable diseases is summarized in Table 1.

## 8. Conclusions

BF is of utmost importance, not only for the adequate and optimal growth of neonates and toddlers but also for its protective effects toward non-communicable disease development in the short and long run. All children benefit from BF in terms of development, health, and survival. It helps develop human capital and saves the lives of women. The advantages are available to people in low-, middle-, and high-income nations. BF offers mothers, children, and society immediate and long-term health, besides economic and environmental benefits. Political backing and monetary investment are required to safeguard, encourage, and support breastfeeding to realize these benefits. Thus, pediatricians should promote BF, support the mother–infant dyad, and consider BM a real “health voucher” that can last lifelong. However, further studies are needed to better define the extent and duration of BF’s protective power in this context and to analyze the relationship between BF and other categories of non-communicable diseases.

## Figures and Tables

**Table 1:** Relationship between BF and non-communicable disease.

Non Communicable Disease	BF Effect on Disease Development
Asthma	There is no direct effect of BF on development of lung function. However, a sort of protection may be secondarily due to a decreased vulnerability to early viral infections or chronic inflammatory conditions during preschool years.
Eczema and allergic rhinitis	The protective effect of BF on these diseases is controversial and may be seen mostly within the first years of life. Specific microbial genes and products (such as human milk oligosaccharides) of human milk have some immunomodulatory effects and may be essential for this protective effect.
Food allergies	BF appears to provide protection against various common childhood allergic diseases but not food allergies.
T1DM	Evidence of a protective effect of BF vs. no BF and of longer vs. shorter BF against T1DM.
Other autoimmune disease	Few studies suggest protective effects of BF against Rheumatoid arthritis and Multiple Sclerosis; no correlation found between BF and JIA, BF, and SLE; not enough studies looking into the effects of BF towards developing autoimmune thyroiditis.
Overweight/obesity	BF may reduce the incidence of weight excess and the longer the duration of BF, the greater the preventive effect.
Blood hypertension	BF has a small protective effect against systolic blood pressure, mostly in childhood, but other factors such as environment and socioeconomic and demographic characteristics cannot be ruled out.
T2DM	The relationship between BF and T2DM incidence is not yet fully understood.
Hypercholesterolemia	BM is richer in cholesterol than formula milk, which may result in the inhibition of endogenous cholesterol. Earlier studies showed BF may be associated with lower cholesterol concentration.
Glucose metabolism	The relationship between BF and T2DM incidence is not yet fully understood. BF may positively affect fasting insulin and HOMA but does not have an effect on fasting glucose.
Cardiac morphology	There is a beneficial association in subjects born prematurely, but there are not any studies about babies born at term.
Celiac disease	BF during the introduction of gluten into the diet, along with an extended duration of breastfeeding, may provide a protective effect against the onset of CD.
Inflammatory bowel disease	BF, by reducing early and late alterations of the microbiota, may play a protective role in the development of IBD.

## Abbreviations

AD	Atopic dermatitis
BF	Breastfeeding
BM	Breast milk
CD	Coeliac disease
CF	Complementary feeding
CVD	Cardiovascular disease
HDL	High-density lipoprotein
HMGCoA	Hydroxymethylglutaryl coenzyme A
HMO	Human milk oligosaccharides
HT	Hashimoto thyroiditis
IBDs	Inflammatory bowel diseases
Ig	Immunoglobulin
JIA	Juvenile idiopathic arthritis
LDL	Low-density lipoprotein
MS	Multiple sclerosis
RA	Rheumatoid arthritis
TC	Total cholesterol
T1DM	Type 1 diabetes mellitus
T2DM	Type 2 diabetes mellitus
VEGF	Vascular endothelial growth factor
WHO	World Health Organization

## References

[1] Meek J.Y., Noble L., Section on Breastfeeding (2022). Policy Statement: Breastfeeding and the Use of Human Milk. Pediatrics.

[2] Victora C.G., Bahl R., Barros A.J.D., Franca G.V.A., Horton S., Krasevec J., Murch S., Sankar M.J., Walker N., Rollins N.C. (2016). Breastfeeding in the 21st century: Epidemiology, mechanisms, and lifelong effect. Lancet.

[3] Critch J.N., Canadian Paediatric Society, Nutrition and Gastroenterology Committee (2014). Nutrition for healthy term infants, six to 24 months: An overview. Paediatr. Child Health.

[4] (2017). Guideline: Protecting, Promoting and Supporting Breastfeeding in Facilities Providing Maternity and Newborn Services.

[5] Vieira Borba V., Sharif K., Shoenfeld Y. (2018). Breastfeeding and autoimmunity: Programing health from the beginning. Am. J. Reprod. Immunol..

[6] Nongonierma A.B., FitzGerald R.J. (2015). Bioactive properties of milk proteins in humans: A review. Peptides.

[7] Barker D.J.P. (2002). EDITORIAL: The developmental origins of adult disease. Eur. J. Epidemiol..

[8] Barker D.J.P. (2007). The origins of the developmental origins theory. J. Intern. Med..

[9] Rollins N.C., Bhandari N., Hajeebhoy N., Horton S., Lutter C.K., Martines J.C., Piwoz E.G., Richter L.M., Victora C.G., Lancet Breastfeeding Series Group (2016). Why invest, and what it will take to improve breastfeeding practices?. Lancet.

[10] Oddy W.H. (2017). Breastfeeding, Childhood Asthma, and Allergic Disease. Ann. Nutr. Metab..

[11] Nagel G., Büchele G., Weinmayr G., Björkstén B., Chen Y.Z., Wang H., Nystad W., Saraclar Y., Bråbäck L., Batlles-Garrido J. (2009). Effect of breastfeeding on asthma, lung function and bronchial hyperreactivity in ISAAC Phase II. Eur. Respir. J..

[12] Pawankar R. (2014). Allergic diseases and asthma: A global public health concern and a call to action. World Allergy Organ. J..

[13] Lodge C.J., Tan D.J., Lau M.X., Dai X., Tham R., Lowe A.J., Bowatte G., Allen K.J., Dharmage S.C. (2015). Breastfeeding and asthma and allergies: A systematic review and meta-analysis. Acta Paediatr..

[14] Lowe A.J., Carlin J.B., Bennett C.M., Abramson M.J., Hosking C.S., Hill D.J., Dharmage S.C. (2006). Atopic disease and breast-feeding—Cause or consequence?. J. Allergy Clin. Immunol..

[15] Wu T.D., Brigham E.P., McCormack M.C. (2019). Asthma in the Primary Care Setting. Med. Clin. N. Am..

[16] Xue M., Dehaas E., Chaudhary N., O’Byrne P., Satia I., Kurmi O.P. (2021). Breastfeeding and risk of childhood asthma: A systematic review and meta-analysis. ERJ Open Res..

[17] Brew B.K., Allen C.W., Toelle B.G., Marks G.B. (2011). Systematic review and meta-analysis investigating breast feeding and childhood wheezing illness. Paediatr. Perinat Epidemiol..

[18] Gorlanova O., Appenzeller R., Mahmoud Y.S., Ramsey K.A., Usemann J., Decrue F., Kuehni C.E., Röösli M., Latzin P., Fuchs O. (2020). Effect of breastfeeding duration on lung function, respiratory symptoms and allergic diseases in school-age children. Pediatr. Pulmonol..

[19] Flohr C., Henderson A.J., Kramer M.S., Patel R., Thompson J., Rifas-Shiman S.L., Yang S., Vilchuck K., Bogdanovich N., Hameza M. (2018). Effect of an Intervention to Promote Breastfeeding on Asthma, Lung Function, and Atopic Eczema at Age 16 Years: Follow-up of the PROBIT Randomized Trial. JAMA Pediatr..

[20] Chiu C.Y., Liao S.L., Su K.W., Tsai M.H., Hua M.C., Lai S.H., Chen L.C., Yao T.C., Yeh K.W., Huang J.L. (2016). Exclusive or Partial Breastfeeding for 6 Months Is Associated With Reduced Milk Sensitization and Risk of Eczema in Early Childhood: The PATCH Birth Cohort Study. Medicine.

[21] Rahman T., Sarwar P.F., Potter C., Comstock S.S., Klepac-Ceraj V. (2023). Role of human milk oligosaccharide metabolizing bacteria in the development of atopic dermatitis/eczema. Front. Pediatr..

[22] Ta L.D.H., Chan J.C.Y., Yap G.C., Purbojati R.W., Drautz-Moses D.I., Koh Y.M., Tay C.J.X., Huang C.-H., Kioh D.Y.Q., Woon J.Y. (2020). A compromised developmental trajectory of the infant gut microbiome and metabolome in atopic eczema. Gut Microbes.

[23] Koukou Z., Papadopoulou E., Panteris E., Papadopoulou S., Skordou A., Karamaliki M., Diamanti E. (2023). The Effect of Breastfeeding on Food Allergies in Newborns and Infants. Children.

[24] Grimshaw K.E.C., Bryant T., Oliver E.M., Martin J., Maskell J., Kemp T., Mills E.N.C., Foote K.D., Margetts B.M., Beyer K. (2015). Incidence and risk factors for food hypersensitivity in UK infants: Results from a birth cohort study. Clin. Transl. Allergy.

[25] Venter C., Pereira B., Voigt K., Grundy J., Clayton C.B., Higgins B., Arshad S.H., Dean T. (2009). Factors associated with maternal dietary intake, feeding and weaning practices, and the development of food hypersensitivity in the infant. Pediatr. Allergy Immunol..

[26] Capra M.E., Decarolis N.M., Monopoli D., Laudisio S.R., Giudice A., Stanyevic B., Esposito S., Biasucci G. (2024). Complementary feeding: Tradition, innovation and pitfalls. Nutrients.

[27] Lachover-Roth I., Cohen-Engler A., Furman Y., Rosman Y., Meir-Shafrir K., Mozer-Mandel M., Farladansky-Gershnabel S., Biron-Shental T., Confino-Cohen R. (2023). Food allergy and infant feeding practices: Are they related?. Ann. Allergy Asthma Immunol..

[28] di Mauro G., Bernardini R., Barberi S., Capuano A., Correra A., Angelis G.L.D., Iacono I.D., de Martino M., Ghiglioni D., Di Mauro D. (2016). Prevention of food and airway allergy: Consensus of the Italian Society of Preventive and Social Paediatrics, the Italian Society of Paediatric Allergy and Immunology, and Italian Society of Pediatrics. World Allergy Organ. J..

[29] Libuda L., Filipiak-Pittroff B., Standl M., Schikowski T., von Berg A., Koletzko S., Bauer C.-P., Heinrich J., Berdel D., Gappa M. (2023). Full Breastfeeding and Allergic Diseases-Long-Term Protection or Rebound Effects?. Nutrients.

[30] Cardwell C.R., Stene L.C., Ludvigsson J., Rosenbauer J., Cinek O., Svensson J., Perez-Bravo F., Memon A., Gimeno S.G., Wadsworth E.J. (2012). Breastfeeding and childhood-onset type 1 diabetes: A pooled analysis of individual participant data from 43 observational studies. Diabetes Care.

[31] Güngör D., Nadaud P., LaPergola C.C., Dreibelbis C., Wong Y.P., Terry N., Abrams S.A., Beker L., Jacobovits T., Järvinen K.M. (2019). Infant milk feeding practices and diabetes outcomes in offspring: A systematic review. Am. J. Clin. Nutr..

[32] Lund-Blix N.A., Sander S.D., Størdal K., Andersen A.-M.N., Rønningen K.S., Joner G., Skrivarhaug T., Njølstad P.R., Husby S., Stene L.C. (2017). Infant Feeding and Risk of Type 1 Diabetes in Two Large Scandinavian Birth Cohorts. Diabetes Care.

[33] Alotiby A.A. (2023). The role of breastfeeding as a protective factor against the development of the immune-mediated diseases: A systematic review. Front. Pediatr..

[34] Lund-Blix N.A., Stene L.C., Rasmussen T., Torjesen P.A., Andersen L.F., Rønningen K.S. (2015). Infant feeding in relation to islet autoimmunity and type 1 diabetes in genetically susceptible children: The MIDIA Study. Diabetes Care.

[35] Holz A., Riefflin M., Heesen C., Riemann-Lorenz K., Obi N., Becher H. (2022). Breastfeeding and Risk of Multiple Sclerosis: A Systematic Review and Meta-Analysis of Observational Studies. Neuroepidemiology.

[36] Ragnedda G., Leoni S., Parpinel M., Casetta I., Riise T., Myhr K.-M., Wolfson C., Pugliatti M. (2015). Reduced duration of breastfeeding is associated with a higher risk of multiple sclerosis in both Italian and Norwegian adult males: The EnvIMS study. J. Neurol..

[37] Conradi S., Malzahn U., Paul F., Quill S., Harms L., Bergh F.T., Ditzenbach A., Georgi T., Heuschmann P., Rosche B. (2013). Breastfeeding is associated with lower risk for multiple sclerosis. Mult. Scler..

[38] Graves J.S., Chitnis T., Weinstock-Guttman B., Rubin J., Zelikovitch A.S., Nourbakhsh B., Simmons T., Waltz M., Casper T.C., Waubant E. (2017). Maternal and Perinatal Exposures Are Associated With Risk for Pediatric-Onset Multiple Sclerosis. Pediatrics.

[39] Kindgren E., Fredrikson M., Ludvigsson J. (2017). Early feeding and risk of Juvenile idiopathic arthritis: A case control study in a prospective birth cohort. Pediatr. Rheumatol. Online J..

[40] Mason T., Rabinovich C.E., Fredrickson D.D., Amoroso K., Reed A.M., Stein L.D., Kredich D.W. (1995). Breastfeeding and the development of juvenile rheumatoid arthritis. J. Rheumatol..

[41] Koker O., Aliyeva A., Sahin S., Adrovic A., Yildiz M., Haslak F., Gunalp A., Barut K., Kasapcopur O. (2022). An overview of the relationship between juvenile idiopathic arthritis and potential environmental risk factors: Do early childhood habits or habitat play a role in the affair?. Int. J. Rheum Dis..

[42] Rosenberg A.M. (1996). Evaluation of associations between breast feeding and subsequent development of juvenile rheumatoid arthritis. J. Rheumatol..

[43] Shenoi S., Shaffer M.L., Wallace C.A. (2016). Environmental Risk Factors and Early-Life Exposures in Juvenile Idiopathic Arthritis: A Case-Control Study. Arthritis Care Res..

[44] Chen H., Wang J., Zhou W., Yin H., Wang M. (2015). Breastfeeding and Risk of Rheumatoid Arthritis: A Systematic Review and Metaanalysis. J. Rheumatol..

[45] Fort P., Moses N., Fasano M., Goldberg T., Lifshitz F. (1990). Breast and soy-formula feedings in early infancy and the prevalence of autoimmune thyroid disease in children. J. Am. Coll. Nutr..

[46] Räisänen L., Viljakainen H., Sarkkola C., Kolho K.-L. (2021). Perinatal risk factors for pediatric onset type 1 diabetes, autoimmune thyroiditis, juvenile idiopathic arthritis, and inflammatory bowel diseases. Eur. J. Pediatr..

[47] Benjamin E.J., Virani S.S., Callaway C.W., Chamberlain A.M., Chang A.R., Cheng S., Chiuve S.E., Cushman M., Delling F.N., Deo R. (2018). Heart Disease and Stroke Statistics—2018 Update: A Report From the American Heart Association. Circulation.

[48] Stone N., Robinson J.G., McBride F.P., Schwartz F.J.S., Shero S.T., Smith S.C., Watson K., Wilson P.W.F., Lichtenstein A.H., Merz C.N.B. (2014). 2013 ACC/AHA Guideline on the Treatment of Blood Cholesterol to Reduce Atherosclerotic Cardiovascular Risk in Adults. Circulation.

[49] Candelino M., Tagi V.M., Chiarelli F. (2022). Cardiovascular risk in children: A burden for future generations. Ital. J. Pediatr..

[50] de Ferranti S.D., Steinberger J., Ameduri R., Baker A., Gooding H., Kelly A.S., Mietus-Snyder M., Mitsnefes M.M., Peterson A.L., St-Pierre J. (2019). Cardiovascular Risk Reduction in High-Risk Pediatric Patients: A Scientific Statement From the American Heart Association. Circulation.

[51] El-Khuffash A., Jain A., Lewandowski A.J., Levy P.T. (2020). Preventing disease in the 21st century: Early breast milk exposure and later cardiovascular health in premature infants. Pediatr. Res..

[52] Capra M.E., Pederiva C., Viggiano C., De Santis R., Banderali G., Biasucci G. (2021). Nutritional approach to prevention and treatment of cardiovascular disease in childhood. Nutrients.

[53] Ballard O., Morrow A.L. (2013). Human milk composition: Nutrients and bioactive factors. Pediatr. Clin. N. Am..

[54] Hui X., Lam K.S.L., Vanhoutte P.M., Xu A. (2012). Adiponectin and cardiovascular health: An update. Br. J. Pharmacol..

[55] Suzuki Y.A., Lopez V., Lönnerdal B. (2005). Mammalian lactoferrin receptors: Structure and function. Cell. Mol. Life Sci..

[56] Hassiotou F., Hartmann P.E. (2014). At the dawn of a new discovery: The potential of breast milk stem cells. Adv. Nutr..

[57] Ounpuu S., Negassa A., Yusuf S. (2001). INTER-HEART: A global study of risk factors for acute myocardial infarction. Am. Heart J..

[58] Gillman M.W., Rifas-Shiman S.L., Camargo C.A., Berkey C.S., Frazier A.L., Rockett H.R., Field A.E., Colditz G.A. (2001). Risk of overweight among adolescents who were breastfed as infants. JAMA.

[59] Horta B.L., Loret de Mola C., Victora C.G. (2015). Long-term consequences of breastfeeding on cholesterol, obesity, systolic blood pressure and type 2 diabetes: A systematic review and meta-analysis. Acta Paediatr..

[60] Owen C.G., Whincup P.H., Gilg J.A., Cook D.G. (2003). Effect of breast feeding in infancy on blood pressure in later life: Systematic review and meta-analysis. BMJ.

[61] Hosaka M., Asayama K., Staessen J.A., Ohkubo T., Hayashi K., Tatsuta N., Kurokawa N., Satoh M., Hashimoto T., Hirose T. (2012). Breastfeeding leads to lower blood pressure in 7-year-old Japanese children: Tohoku Study of Child Development. Hypertens. Res..

[62] Liu J., Gao D., Li Y., Chen M., Wang X., Ma Q., Ma T., Chen L., Ma Y., Zhang Y. (2022). Breastfeeding Duration and High Blood Pressure in Children and Adolescents: Results from a Cross-Sectional Study of Seven Provinces in China. Nutrients.

[63] de Jonge L.L., van Osch-Gevers L., Geelhoed J.M., Hofman A., Steegers E.A., Helbing W.A., Jaddoe V.W. (2010). Breastfeeding is not associated with left cardiac structures and blood pressure during the first two years of life. The Generation R Study. Early Hum. Dev..

[64] Järvisalo M.J., Hutri-Kähönen N., Juonala M., Mikkilä V., Räsänen L., Lehtimäki T., Viikari J., Raitakari O.T. (2008). Breast feeding in infancy and arterial endothelial function later in life. The Cardiovascular Risk in Young Finns Study. Eur. J. Clin. Nutr..

[65] Holmes V.A., Cardwell C., McKinley M.C., Young I.S., Murray L.J., Boreham C.A., Woodside J.V. (2010). Association between breast-feeding and anthropometry and CVD risk factor status in adolescence and young adulthood: The Young Hearts Project, Northern Ireland. Public Health Nutr..

[66] Rudnicka A.R., Owen C.G., Strachan D.P. (2007). The effect of breastfeeding on cardiorespiratory risk factors in adult life. Pediatrics.

[67] Wilson P.W., D’Agostino R.B., Levy D., Belanger A.M., Silbershatz H., Kannel W.B. (1998). Prediction of coronary heart disease using risk factor categories. Circulation.

[68] Owen C.G., Whincup P.H., Kaye S.J., Martin R.M., Davey Smith G., Cook D.G., Bergstrom E., Black S., Wadsworth M.E., Fall C.H. (2008). Does initial breastfeeding lead to lower blood cholesterol in adult life? A quantitative review of the evidence. Am. J. Clin. Nutr..

[69] Capra M.E., Monopoli D., Decarolis N.M., Giudice A., Stanyevic B., Esposito S., Biasucci G. (2023). Dietary Models and Cardiovascular Risk Prevention in Pediatric Patients. Nutrients.

[70] Li Y., Gao D., Chen L., Ma T., Ma Y., Chen M., Dong B., Dong Y., Ma J., Arnold L. (2021). The Association between Breastfeeding Duration and Lipid Profile among Children and Adolescents. Nutrients.

[71] Hui L.L., Kwok M.K., Nelson E.A.S., Lee S.L., Leung G.M., Schooling C.M. (2019). Breastfeeding in Infancy and Lipid Profile in Adolescence. Pediatrics.

[72] Singhal A., Cole T.J., Fewtrell M., Lucas A. (2004). Breastmilk feeding and lipoprotein profile in adolescents born preterm: Follow-up of a prospective randomised study. Lancet.

[73] Mayer-Davis E.J., Lawrence J.M., Dabelea D., Divers J., Isom S., Dolan L., Imperatore G., Linder B., Marcovina S., Pettitt D.J. (2017). Incidence Trends of Type 1 and Type 2 Diabetes among Youths, 2002–2012. N. Engl. J. Med..

[74] Cheshmeh S., Nachvak S.M., Hojati N., Elahi N., Heidarzadeh-Esfahani N., Saber A. (2022). The effects of breastfeeding and formula feeding on the metabolic factors and the expression level of obesity and diabetes-predisposing genes in healthy infants. Physiol. Rep..

[75] Boddicker R.L., Koltes J.E., Fritz-Waters E.R., Koesterke L., Weeks N., Yin T., Mani V., Nettleton D., Reecy J.M., Baumgard L.H. (2016). Genome-wide methylation profile following prenatal and postnatal dietary omega-3 fatty acid supplementation in pigs. Anim. Genet..

[76] Hui L.L., Kwok M.K., Nelson E.A.S., Lee S.L., Leung G.M., Schooling C.M. (2018). The association of breastfeeding with insulin resistance at 17 years: Prospective observations from Hong Kong’s “Children of 1997” birth cohort. Matern. Child Nutr..

[77] Pirilä S., Taskinen M., Viljakainen H., Mäkitie O., Kajosaari M., Saarinen-Pihkala U.M., Turanlahti M. (2014). Breast-fed infants and their later cardiovascular health: A prospective study from birth to age 32 years. Br. J. Nutr..

[78] Lewandowski A.J., Levy P.T., Bates M.L., McNamara P.J., Nuyt A.M., Goss K.N. (2020). Impact of the Vulnerable Preterm Heart and Circulation on Adult Cardiovascular Disease Risk. Hypertension.

[79] Lyons K.E., Ryan C.A., Dempsey E.M., Ross R.P., Stanton C. (2020). Breast milk, a source of beneficial microbes and associated benefits for infant health. Nutrients.

[80] Houghteling P.D., Walker W.A. (2015). Why is initial bacterial colonization of the intestine important to infants’and children’s health?. J. Pediatr. Gastroenterol. Nutr..

[81] Milani C., Duranti S., Bottacini F., Casey E., Turroni F., Mahony J., Belzer C., Delgado Palacio S., Arboleya Montes S., Mancabelli L. (2017). The first microbial colonizers of the human gut: Composition, activities, and health implications of the infant gut Microbiota. Microbiol. Mol. Biol. Rev..

[82] Henrick B.M., Rodriguez L., Lakshmikanth T., Pou C., Henckel E., Arzoomand A., Olin A., Wang J., Mikes J., Tan Z. (2021). Bifidobacteria mediated immune system imprinting early in life. Cell.

[83] Doare L., Holder K., Bassett B., Pannaraj A. (2018). Mother’s milk: A purposeful contribution to the development of the infant Microbiota and immunity. Front. Immunol..

[84] Chong H.-Y., Tan L.T.-H., Law J.W.-F., Hong K.-W., Ratnasingam V., Ab Mutalib N.-S., Lee L.-H., Letchumanan V. (2022). Exploring the potential of human milk and formula milk on infants’ gut and health. Nutrients.

[85] Hermansson H., Kumar H., Collado M.C., Salminen S., Isolauri E., Rautava S. (2019). Breast milk Microbiota is shaped by mode of delivery and intrapartum antibiotic exposure. Front. Nutr..

[86] Cabrera-Rubio R., Collado M.C., Laitinen K., Salminen S., Isolauri E., Mira A. (2012). The human milk microbiome changes over lactation and is shaped by maternal weight and mode of delivery. Am. J. Clin. Nutr..

[87] An Den Elsen L., Rekima A., Verhasselt V. (2019). Early-Life Nutrition and Gut Immune Development.

[88] Narasimhan P.B., Marcovecchio P., Hamers A.A.J., Hedrick C.C. (2019). Nonclassical monocytes in health and disease. Annu. Rev. Immunol..

[89] Gu J., Ni X., Pan X., Lu H., Lu Y., Zhao J., Zheng S.G., Hippen K.L., Wang X., Lu L. (2017). Human CD39hi regulatory T cells present stronger stability and function under inflammatory conditions. Cell. Mol. Immunol..

[90] Ehrlich A.M., Pacheco A.R., Henrick B.M., Taft D., Xu G., Huda M.N., Mishchuk D., Goodson M.L., Slupsky C., Barile D. (2020). Indole-3-lactic acid associated with Bifidobacterium-dominated microbiota significantly decreases inflammation in intestinal epithelial cells. BMC Microbiol..

[91] Davis E.C., Castagna V.P., Sela D.A., Hillard M.A., Lindberg S., Mantis N.J., Seppo A.E., Järvinen K.M. (2022). Gut microbiome and breast-feeding: Implications for early immune development. J. Allergy Clin. Immunol..

[92] Praveen P., Jordan F., Priami C., Morine M.J. (2015). The role of breast-feeding in infant immune system: A systems perspective on the intestinal microbiome. Microbiome.

[93] Silano M., Agostoni C., Sanz Y., Guandalini S. (2016). Infant feeding and risk of developing celiac disease: A systematic review. BMJ Open.

[94] Ivarsson A., Hernell O., Stenlund H., Persson L.Å. (2002). Breast-feeding protects against celiac disease. Am. J. Clin. Nutr..

[95] Norris J.M., Barriga K., Hoffenberg E.J., Taki I., Miao D., Haas J.E., Emery L.M., Sokol R.J., Erlich H.A., Eisenbarth G.S. (2005). Risk of celiac disease autoimmunity and timing of gluten introduction in the diet of infants at increased risk of disease. JAMA.

[96] Agostoni C., Braegger C., Decsi T., Kolacek S., Koletzko B., Michaelsen K.F., Mihatsch W., Moreno L.A., Puntis J., ESPGHAN Committee on Nutrition (2009). Breast-feeding: A commentary by the ESPGHAN Committee on Nutrition. J. Pediatr. Gastroenterol. Nutr..

[97] Ananthakrishnan A.N., Bernstein C.N., Iliopoulos D., Macpherson A., Neurath M.F., Ali R.A.R., Vavricka S.R., Fiocchi C. (2018). Environmental triggers in IBD: A review of progress and evidence. Nat. Rev. Gastroenterol. Hepatol..

[98] Agrawal M., Sabino J., Frias-Gomes C., Hillenbrand C.M., Soudant C., Axelrad J.E., Shah S.C., Ribeiro-Mourão F., Lambin T., Peter I. (2021). Early life exposures and the risk of inflammatory bowel disease: Systematic review and meta-analyses. EClinicalMedicine.

[99] Azad M.B., Konya T., Maughan H., Guttman D.S., Field C.J., Chari R.S., Sears M.R., Becker A.B., Scott J.A., Kozyrskyj A.L. (2013). Gut microbiota of healthy Canadian infants: Profiles by mode of delivery and infant diet at 4 months. CMAJ.

[100] Azad M., Konya T., Persaud R., Guttman D., Chari R., Field C., Sears M.R., Mandhane P., Turvey S., Subbarao P. (2016). Impact of maternal Intrapartum antibiotics, method of birth and breastfeeding on gut microbiota during the first year of life: A prospective cohort study. BJOG.

[101] Madsen K.L., Fedorak R.N., Tavernini M.M., Doyle J.S. (2002). Normal breast milk limits the development of colitis in IL-10-deficient mice. Inflamm. Bowel Dis..

[102] Barclay A.R., Russell R.K., Wilson M.L., Gilmour W.H., Satsangi J., Wilson D.C. (2009). Systematic review: The role of breastfeeding in the development of pediatric inflammatory bowel disease. J. Pediatr..

